# Supplementary material for: Core outcome sets in symptomatic peripheral artery disease, COS-PAD: Study protocol for developing core outcome sets in symptomatic PAD utilising systematic reviews, interviews, and delphi consensus
Source: PLoS One. 2025 Jul 17;20(7):e0328453. doi: 10.1371/journal.pone.0328453 (PMC12270175; doi:10.1371/journal.pone.0328453)
Supplement: S1 Table — (DOCX) [file pone.0328453.s001.docx]

**Supplementary Materials**

**Table S1.** Search strategy of the Reported Outcomes in Studies of Intermittent Claudication Systematic Review, conducted in Medline via Ovid

| Item number | Search Item |
| --- | --- |
| 1 | Intermittent Claudication/ |
| 2 | claudic*.mp. |
| 3 | exp Peripheral Vascular Diseases/ |
| 4 | Arterial Occlusive Diseases/ |
| 5 | Peripheral Arterial Disease/ |
| 6 | 1 or 2 or 3 or 4 or 5 |
| 7 | randomized controlled trial.pt. |
| 8 | controlled clinical trial.pt. |
| 9 | clinical trial.pt. |
| 10 | comparative study.pt. |
| 11 | randomized.ab. |
| 12 | placebo.ab. |
| 13 | clinical trials as topic.sh. |
| 14 | randomly.ab. |
| 15 | trial.ti. |
| 16 | 7 or 8 or 9 or 11 or 12 or 13 or 14 or 15 |
| 17 | exp animals/ not humans.sh. |
| 18 | 16 not 17 |
| 19 | 6 and 18 |
| 20 | limit 19 to english language |
