# Supplementary material for: Core outcome sets in symptomatic peripheral artery disease, COS-PAD: Study protocol for developing core outcome sets in symptomatic PAD utilising systematic reviews, interviews, and delphi consensus
Source: PLoS One. 2025 Jul 17;20(7):e0328453. doi: 10.1371/journal.pone.0328453 (PMC12270175; doi:10.1371/journal.pone.0328453)
Supplement: S2 Table — (DOCX) [file pone.0328453.s002.docx]

**Supplementary Materials**

| Item number | Search Item |
| --- | --- |
| 1 | intermittent claudication/ |
| 2 | "claudic*".af. |
| 3 | peripheral vascular disease/ |
| 4 | peripheral occlusive artery disease/ |
| 5 | 1 or 2 or 3 or 4 |
| 6 | Randomized controlled trial/ |
| 7 | Controlled clinical study/ |
| 8 | random$.ti,ab. |
| 9 | randomization/ |
| 10 | placebo.ti,ab. |
| 11 | trial.ti. |
| 12 | or/6-11 |
| 13 | (random$ adj sampl$ adj7 ("cross section$" or questionnaire$1 or survey$ or database$1)).ti,ab. not (comparative study/ or controlled study/ or randomi?ed controlled.ti,ab. or randomly assigned.ti,ab.) |
| 14 | Cross-sectional study/ not (randomized controlled trial/ or controlled clinical study/ or controlled study/ or randomi?ed controlled.ti,ab. or control group$1.ti,ab.) |
| 15 | (((case adj control$) and random$) not randomi?ed controlled).ti,ab. |
| 16 | (Systematic review not (trial or study)).ti. |
| 17 | (nonrandom$ not random$).ti,ab. |
| 18 | "Random field$".ti,ab. |
| 19 | (review.ab. and review.pt.) not trial.ti. |
| 20 | "we searched".ab. and (review.ti. or review.pt.) |
| 21 | "update review".ab. |
| 22 | (databases adj4 searched).ab. |
| 23 | (rat or rats or mouse or mice or swine or porcine or murine or sheep or lambs or pigs or piglets or rabbit or rabbits or cat or cats or dog or dogs or cattle or bovine or monkey or monkeys or trout or marmoset$1).ti. and animal experiment/ |
| 24 | Animal experiment/ not (human experiment/ or human/) |
| 25 | or/13-24 |
| 26 | 12 not 25 |
| 27 | 5 and 26 |
| 28 | limit 27 to (english language and yr="2015 -Current") |

**Table 2.** Search strategy of the Reported Outcomes in Studies of Intermittent Claudication Systematic Review, conducted in Embase via Ovid
