# Supplementary material for: Core outcome sets in symptomatic peripheral artery disease, COS-PAD: Study protocol for developing core outcome sets in symptomatic PAD utilising systematic reviews, interviews, and delphi consensus
Source: PLoS One. 2025 Jul 17;20(7):e0328453. doi: 10.1371/journal.pone.0328453 (PMC12270175; doi:10.1371/journal.pone.0328453)
Supplement: S3 Table — (DOCX) [file pone.0328453.s003.docx]

**Supplementary Materials**

**Table 3.** Search strategy of the Reported Outcomes in Studies of Chronic Limb Threatening Ischaemia Systematic Review.

| Ovid MEDLINE(R) ALL <1946 to February 07, 2024>  Run 7th February 2024  1 ((limb* or leg*) adj3 isch?emi*).ti,kw. 6429  2 CLI.tw. 3021  3 CLTI.tw. 535  4 "limb* threat*".ti,kw. 645  5 SLI.tw. 2297  6 Chronic Limb-Threatening Ischemia/ 267  7 1 or 2 or 3 or 4 or 5 or 6 10703  Embase <1974 to 2024 February 07>  Run 7th February 2024  1 ((limb* or leg*) adj3 isch?emi*).ti,kw. 9033  2 CLI.tw. 5147  3 CLTI.tw. 793  4 "limb* threat*".ti,kw. 829  5 SLI.tw. 2630  6 *critical limb ischemia/ 3232  7 1 or 2 or 3 or 4 or 5 or 6 15218  Cochrane Central Register of Controlled Trials (CENTRAL)  Date Run: 7th February 2024 13:59:42  ID Search Hits  #1 ((limb* OR leg*) NEAR/3 isch?emi*):ti,kw 1454  #2 CLI:ti,ab 555  #3 CLTI:ti,ab 49  #4 (limb* NEXT threat*):ti,kw 60  #5 SLI:ti,ab 156  #6 MeSH descriptor: [Chronic Limb-Threatening Ischemia] this term only 8  #7 {OR #1-#6} 1798 (1775 from CENTRAL)  CINAHL – Run 7th February 2024  S1 ((limb* OR leg*) N3 isch?emi*) 426  S2 CLI 585  S3 CLTI 85  S4 limb* N1 threat* 678  S5 SLI NOT specific language impairment 188  S6 (MH "Chronic Limb-Threatening Ischemia") 78  S7 S1 OR S2 OR S3 OR S4 OR S5 OR S6 1,781 |
| --- |
